# Elements of Human Physiology

**Published:** 1884-09

**Authors:** 


**Elements of Human Physiology.** By Henry Powers, M. B., F. R. C. S., St. Bartholomew's Hospital, London. Illustrated with 47 engravings. Philadelphia: H. C. Lea's Son & Co.

